# Identification of a 7‐microRNA signature in plasma as promising biomarker for nasopharyngeal carcinoma detection

**DOI:** 10.1002/cam4.2676

**Published:** 2019-12-19

**Authors:** Huo Zhang, Xuan Zou, Lirong Wu, Shiyu Zhang, Tongshan Wang, Ping Liu, Wei Zhu, Jun Zhu

**Affiliations:** ^1^ Department of Oncology First Affiliated Hospital of Nanjing Medical University Nanjing Jiangsu PR China; ^2^ Department of Medical Oncology Northern Jiangsu People's Hospital Affiliated to Yangzhou University Yangzhou Jiangsu PR China; ^3^ Fudan University Shanghai Cancer Center Xuhui Shanghai PR China; ^4^ Department of Radiation Oncology Nanjing Medical University Affiliated Cancer Hospital Jiangsu Cancer Hospital Jiangsu Institute of Cancer Research Nanjing PR China

**Keywords:** biomarker, nasopharyngeal carcinoma, plasma miRNA, qRT‐PCR

## Abstract

**Background:**

Circulating microRNAs (miRNAs) have become reliable sources of non‐invasive biomarkers for cancer diagnosis. Identification of promising miRNA biomarkers in plasma might benefit a lot to the detection of nasopharyngeal carcinoma (NPC).

**Methods:**

The Exiqon miRNA qPCR panel was used in the screening stage to identify candidate miRNAs, which were further verified by quantitative reverse transcription polymerase chain reaction (qRT‐PCR) in the following three stages among plasma samples from 200 NPC patients and 189 healthy donors (as normal controls [NCs]). The identified miRNAs were further explored in tissue specimens (48 NPC vs 32 NCs) and plasma exosomes (32 NPC vs 32 NCs). Survival analyses were ultimately conducted by Cox regression models and Kaplan‐Meier curves using log‐rank tests.

**Results:**

We identified a 7‐miRNA signature including let‐7b‐5p, miR‐140‐3p, miR‐144‐3p, miR‐17‐5p, miR‐20a‐5p, miR‐20b‐5p, and miR‐205‐5p in plasma for NPC diagnosis after four‐stage validation. The areas under the receiver operating characteristic curve (AUCs) for the signature were 0.879, 0.884, 0.921, and 0.807 for the training, testing, external validation stage, and the combined three stages, respectively. In NPC tissues, miR‐144‐3p, miR‐17‐5p, miR‐20a‐5p, and miR‐205‐5p were consistently up‐regulated while let‐7b‐5p and miR‐140‐3p were significantly down‐regulated compared to NCs. However, none of the seven identified miRNAs were dysregulated in plasma‐derived exosomes in NPC patients. As to survival analysis, none of the seven miRNAs seemed to be associated with NPC prognosis.

**Conclusion:**

We identified a 7‐miRNA signature in plasma as promising non‐invasive biomarkers for NPC detection.

## Graphical abstract

Circulating microRNAs have become reliable sources of non‐invasive biomarkers for cancer diagnosis. We identified a 7‐miRNA signature in plasma as promising non‐invasive biomarkers for NPC detection.

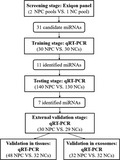

## 1 INTRODUCTION

Nasopharyngeal carcinoma (NPC) is a kind of uncommon cancer derived from the epithelium of nasopharynx.^1^ Although compared to other cancers, NPC is rarer with the proportion of only about 0.6% of all diagnosed cancers worldwide, it has relatively higher incidence rate in some specific ethnic populations and regions such as the east and southeast parts of Asia and other less developed areas.^2^ During the past decades, incidence and mortality rate of NPC have decreased gradually due to effective screening and treatment strategies including radiotherapy, combined chemotherapy, and the emerging immunotherapy (such as adoptive T‐cell transfer and immune checkpoint inhibitor).^3^, ^4^, ^5^ However, it is still a heavy health burden in China which needs enhanced control and prevention.^6^ NPC, especially the undifferentiated form (WHO type III) which represents almost 80% of all the cases, has shown consistent association with Epstein‐Barr virus (EBV) infection.^7^, ^8^, ^9^ Various studies have confirmed the diagnostic value of circulating cancer‐derived EBV DNA as biomarker for NPC early detection.^10^, ^11^ Antibodies in response to different EBV antigenic elements can be released into body fluids (serum and saliva) and also form potential screening biomarkers for NPC diagnosis.^12^, ^13^ Although these markers may be a help for the confirmation of highly suspected NPC patients, their abilities to screen asymptomatic patients were limited due to unstable sensitivity and specificity.^14^ Therefore, more researches are still in need for the discovery of novel non‐invasive biomarkers to identify NPC patients.

MicroRNAs (miRNAs) are families of small non‐coding RNAs with the length of about 19‐25 nucleotides which function in post‐transcriptional regulation by targeting mRNA and mediating mRNA degradation.^15^ The importance of miRNAs in cancer biology has been underlined by many studies with accumulating evidence in recent years.^16^ Dysregulated miRNAs that exist stably in tumor tissues and peripheral blood circulation are reliable sources of diagnostic or prognostic markers for various cancer types.^17^ For NPC, miRNA expression profiling in tumor tissues has been assessed by a number of studies, but systematic analyses of plasma miRNAs are still inadequate and inconsistent.^18^, ^19^, ^20^

In this study, to identify potential biomarkers for NPC diagnosis, we conducted miRNA profiling in plasma with four‐stage validation by quantitative reverse transcription polymerase chain reaction (qRT‐PCR). MiRNA expression patterns in tissue specimens and plasma‐derived exosomes samples were also analyzed for further exploration.

## 2 MATERIALS AND METHODS

### 2.1 Participants and samples

All the participants (200 NPC patients and 189 healthy donors) were recruited from First Affiliated Hospital of Nanjing Medical University and Jiangsu Cancer Hospital from 2014 to 2016. Patients were all histopathologically confirmed NPC cases. Clinical and histopathological features including gender, age, TNM stage, EBV infection history, and pathological type for each participant were recorded in detail. The study was approved by the Institutional Review Boards of the First Affiliated Hospital of Nanjing Medical University and Jiangsu Cancer Hospital. Informed consent has been obtained from every participant.

Whole venous blood sample was drawn from each participant before any clinical intervention or treatment such as radiotherapy and surgical operation. Samples were initially collected in ethylenediaminetetraacetic acid (EDTA)‐containing tubes (Becton, Dickinson and Company) followed by a two‐step centrifugal process (350 RCF [reactive centrifugal force] for 10 minutes and 20 000 RCF for 10 minutes [Beckman Coulter]) to isolate cell‐free plasma samples within 12 hours. The obtained plasma samples were then restored in RNase‐free tube at −80°C ready for future analysis. In all, we collected 200 plasma samples from NPC patients and 189 plasma samples that were set as normal controls (NCs) from healthy donors. Additional 48 frozen tumor tissue specimens from NPC patients undergoing surgery and 32 paraffin‐embedded nasal mucosa tissue specimens from healthy donors were also collected and kept in liquid nitrogen for further exploration.

### 2.2 Exosomes isolation

We used ExoQuick Exosome Precipitation Solution (System Biosciences, Mountain View, Calif) to isolate exosomes from plasma samples. Following manufacturer's protocols, exosomes pellets were precipitated from the mixture of 400 μL plasma and 100 μL ExoQuick exosome precipitation solution and lysed into 200 μL of RNase‐free water for future RNA extraction. A total of 64 plasma‐derived exosomes samples (32 NPC vs 32 NCs) were collected.

### 2.3 Study design

**Figure 1:**
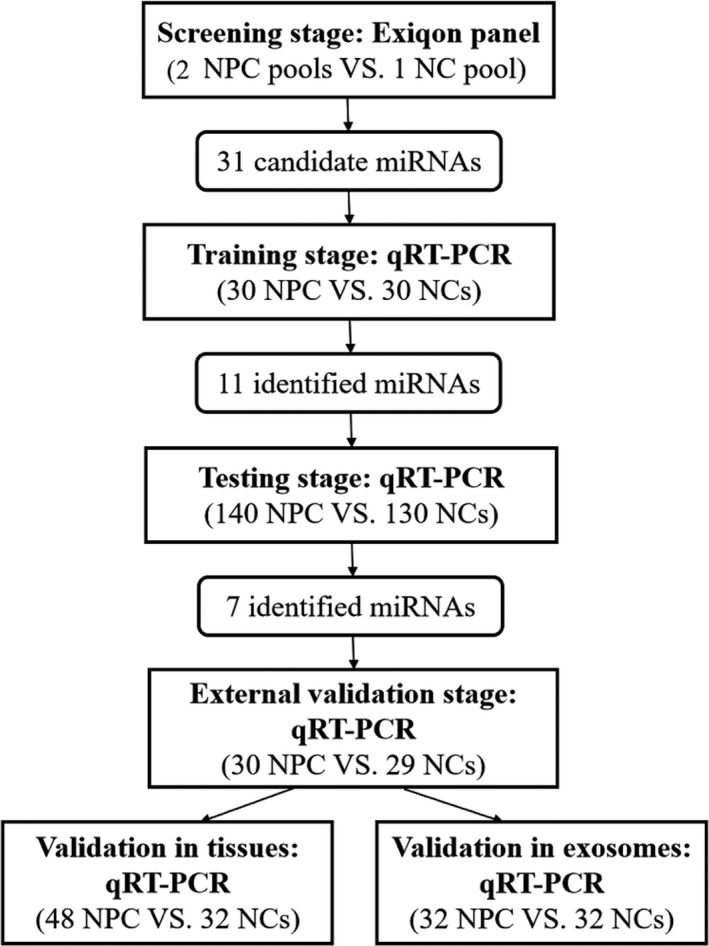
Flow chart of experiment design. (NPC: nasopharyngeal carcinoma; NC: normal control.)

The study was designed into four‐stages: the screening, training, testing, and external validation stages to identify potential miRNA biomarkers for NPC diagnosis. The flow chart of experiment design is given in Figure 1. Several factors were taken into account in sample selection and distribution in the four stages: (a) the purpose of each separate stage (b) the sequence of sample collection in practical operation (c) the balance of non‐experimental factors among four separate sets such as gender, age, TNM stage, and pathological type (d) the basic principal of experiment design—control, randomization, replication, and balance. In the initial screening stage, the miRNA profiling platform‐Exiqon miRCURY‐Ready‐to‐Use PCR‐Human‐panel‐I+II‐ V1.M (Exiqon miRNA qPCR panel, Vedbaek, Denmark; 168 miRNAs) was applied for the selection of candidate miRNAs. We constructed 2 NPC pools and 1 NC pool with per 10 plasma samples being gathered into 1 pooled samples and tried to identify differently expressed miRNAs between NPC and NC pools using the Exiqon miRNA qPCR panel on 7900HT real‐time PCR system (Applied Biosystems). The process was in accordance with the previous study.^21^ Candidate miRNAs were then subjected to following multiple‐stage validation process and analyzed by qRT**‐**PCR among 200 NPC and 189 NCs plasma samples (30 NPC vs 30 NCs for the training stage, 140 NPC vs 130 NCs for the testing stage and 30 NPC vs 29 NCs for the external validation stage). Analysis in the training, testing, and external validation stages was conducted in an orderly and independent manner. The three independent stages were designed for preliminary analysis of candidate miRNAs, accurate verification among larger cohorts and further verification of the established model, respectively. A large sample size in the testing phase was of the greatest importance, since it was the most critical step in determining the final models. At each stage, the number of control samples should match with that of tumor samples to the most extent. In addition, factors such as gender, age, TNM stage, and pathological type were as evenly distributed across four sets as possible to avoid selection bias.

In addition, expression levels of the identified miRNAs in tissue specimens (48 NPC vs 32 NCs) and plasma‐derived exosomes samples (32 NPC vs 32 NCs) were also analyzed by qRT**‐**PCR for further exploration.

### 2.4 RNA extraction

We extracted total RNA using the mirVana PARIS Kit (Ambion) for 200 μL plasma and exosomes samples and Trizol (Invitrogen, Carlsbad, CA, USA) for tissue specimens following manufacturer's instructions. The acquired total RNA was lysed into 100 μL RNase‐free water and kept at 80°C until analysis. The ultraviolet spectrophotometer was applied to evaluate the concentration and purity of total RNA samples. If the concentration of total RNA was less than 10 ng/μL, it was not included in data analysis. During the process, additional 5 μL of synthetic *Caenorhabditis elegans miR‐39* (5nM/L, RiboBio, Guangzhou, China) was added to each sample after denaturing solution (Ambion) for sample‐to‐sample normalization.

### 2.5 Quantitative reverse transcription polymerase chain reaction (qRT‐PCR)

MiRNAs were amplified using Bulge‐Loop^TM^ miRNA qRT‐PCR Primer Set (RiboBio) with specific primers of reverse transcription (RT) and polymerase chain reaction (PCR). According to the previous study, RT and PCR procedures were performed on 7900HT real‐time PCR system (Applied Biosystems) in the condition of 42°C for 60 minutes followed by 70°C for 10 minutes (for RT) and 95°C for 20 seconds, followed by 40 cycles of 95°C for 10 seconds, 60°C for 20 seconds and then 70°C for 10 seconds (for PCR), respectively.^22^ SYBR Green (SYBR® Premix Ex TaqTM II, TaKaRa) was used to calculate the amount of PCR products by the level of fluorescence and melting analysis was introduced to evaluate the specificity of PCR products. As described previously, miRNA expression levels were determined using the 2^−ΔΔCt^ method with *cel‐miR‐39* and *RNU6B (U6,* for tissue samples*)* as reference.

### 2.6 Statistical analysis

Mann‐Whitney *U* test was used to assess the difference of miRNA expression in plasma, exosomes, and tissue specimens between NPC and NC groups. One‐way ANOVA or χ^2^ test was applied to analyze the demographic and clinical characteristics of participants along with their association with miRNA expression patterns. Binary logistic regression analysis was conducted to combine the identified miRNAs into a comprehensive panel. A formula of log distribution was built based on the relative expression data generated from all the 200 NPC patients and 189 NCs: Logit(P) = ln(P/(1‐P)), where P (P = 1/(1 + e‐Logit(P)) implies the probability of identifying the disease case correctly. The predicted probability of being diagnosed as NPC was used to fit receiver operating characteristic (ROC) curves. The area under the ROC curve (AUC) was calculated to estimate the diagnostic performance of individual miRNAs and the constructed panel. The corresponding prognostic value was evaluated by overall survival (OS) rate. Cox's regression models were applied to assess factors related to the OS and Kaplan‐Meier curves using log‐rank tests were used to estimate the association between identified miRNAs and NPC prognosis. SPSS22.0 software (SPSS Inc) and GraphPad Prism 7 (GraphPad Software) were applied for statistical analysis and graph construction. A two‐sided *P*‐value <.05 was considered to be of statistical significance.

## 3 RESULTS

### 3.1 Description of study subjects

A total of 200 NPC patients and 189 NCs which were divided into three independent parts (the training, testing, and external validation stages) were enrolled in this study for the comparison of miRNA expression levels in plasma. Their characteristics are presented in Table 1 and the flow chart of experiment design is shown in Figure 1. No significant difference of gender and age distribution was observed between the case and the control groups. (*P* > .05).

**Table 1:** Demographic and clinical characteristics of NPC patients and NCs

Variables	Training stage		Testing stage		External validation stage	
	Cases (%)	Control (%)	Cases (%)	Control (%)	Cases (%)	Controls (%)
Number	30	30	140	130	30	29
Gender						
Man	22 (73.3)	16 (53.3)	107 (76.4)	82 (63.1)	25 (83.3)	18 (62.1)
Woman	8 (26.7)	14 (46.7)	33 (36.9)	48 (36.9)	5 (16.7)	11 (27.9)
Age						
<65	27 (90.0)	17 (56.7)	116 (82.9)	96 (73.8)	24 (80.0)	17 (58.6)
≥65	3 (10.0)	13 (43.3)	24 (17.1)	34 (26.2)	6 (20.0)	12 (41.4)
TNM stage						
I	0 (0.0)		3 (2.1)		0 (0.0)	
II	6 (20.0)		30 (21.4)		7 (23.3)	
III	14 (46.7)		71 (50.7)		12 (40.0)	
IVa	10 (33.3)		31 (22.1)		9 (30.0)	
IVb	0 (0.0)		5 (3.6)		2 (6.7)	
EBV history						
＜5.00E+02	28 (93.3)		75 (53.6)		2 (6.7)	
≥5.00E+02	2 (6.6)		63 (45.0)		27 (90.0)	
NA	0 (0.0)		2 (1.4)		1 (3.3)	
Pathological type						
PDSCC	26 (86.7)		112 (80.0)		18 (60.0)	
Non‐keratinizing carcinoma	0 (0.0)		9 (6.4)		4 (13.3)	
NA	4 (13.3)		19 (13.6)		8 (26.7)	

Abbreviations: EBV, Epstein‐Barr virus; NA, not available; NCs, normal controls; NPC, nasopharyngeal carcinoma; PDSCC, poorly differentiated squamous cell carcinoma.

### 3.2 Discovery of candidate miRNAs in the screening stage

In the initial screening stage, the Exiqon miRCURY‐Ready‐to‐Use PCR‐Human‐panel‐I+II‐V1.M—a miRNA profiling platform—was employed for the selection of candidate miRNAs in plasma among 2 NPC pools and 1 NC pool. A total of 168 miRNAs with relatively higher expression abundance in plasma/serum were analyzed twice on 384‐well plates by qRT‐PCR. MiRNAs satisfying all of the three following standards were determined to be candidate miRNAs: (a) cycle threshold (Ct) value <37 (b) Ct value being 5 lower than negative control (No Template Control, NTC) (c) expression level being altered with >1.5‐fold or <0.67‐fold in any NPC pool compared to NC pool.^23^ As a result, 31 plasma miRNAs with 25 up‐regulated and 6 down‐regulated were found to be differently expressed between NPC pools and NC pool, which were submitted to further validation in the following three stages (Table S1).

### 3.3 Validation of candidate miRNAs by qRT‐PCR

**Figure 2:**
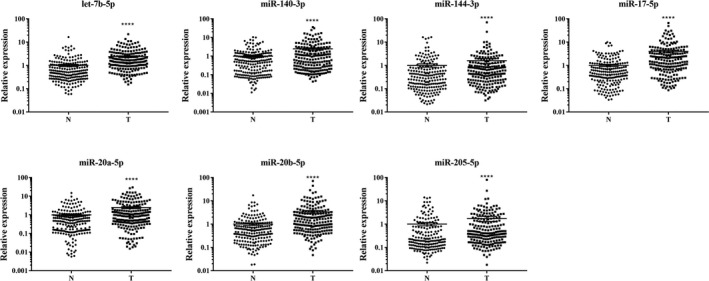
Expression levels of the seven identified plasma miRNAs in combined training, testing, and external validation stages (200 NPC vs 189 NCs). (Y axis represents the relative expression (2^−ΔΔCt^); Horizontal line: mean with 95% CI; N: normal control; T: tumor; **** *P*‐value < .0001.)

The 31 candidate miRNAs were firstly analyzed among plasma samples from 30 NPC patients and 30 NCs in the training stage by qRT‐PCR. Only 11 miRNAs (let‐7b‐5p, miR‐130a‐3p, miR‐140‐3p, miR‐144‐3p, miR‐17‐5p, miR‐20b‐5p, miR‐320c, miR‐660‐5p, miR‐205‐5p, miR‐451a, and miR‐20a‐5p) showed consistent up‐regulation tendency (fold change (FC) >1.5) in NPC patients compared to NCs as in the screening stage. The results were reevaluated in a larger cohort of 140 NPC and 130 NCs serum samples in the following testing stage by qRT‐PCR. In this stage, seven miRNAs (let‐7b‐5p, miR‐140‐3p, miR‐144‐3p, miR‐17‐5p, miR‐20a‐5p, miR‐20b‐5p, and miR‐205‐5p) of the 11 were determined to be consistently up‐regulated in NPC patients (FC >1.5). The seven miRNAs were further validated in the ultimate external validation stage (30 NPC vs 29 NCs) and conclusion remained the same (Table 2). When the three stages were combined, expression patterns of the seven plasma miRNAs showed significant difference between NPC patients and NCs with *P < *.05 (Figure 2).

**Table 2:** Expression levels of the identified 7 miRNAs in the three independent stages (presented as mean ± SD; ΔCT, relative to cel‐miR‐39)

miRNA	Training stage				Testing stage				External validation stage			
	NPC	NC	FC	*P*‐value	NPC	NC	FC	*P*‐value	NPC	NC	FC	*P*‐value
let‐7b‐5p	3.17 ± 0.69	4.30 ± 0.82	2.19	<.001	2.86 ± 1.51	4.70 ± 1.37	3.59	<.001	3.70 ± 0.93	5.16 ± 0.70	2.76	<.001
miR‐140‐3p	6.00 ± 1.16	6.63 ± 1.46	1.54	.034	4.72 ± 2.14	6.54 ± 1.81	3.54	<.001	7.58 ± 0.72	8.35 ± 0.80	1.71	<.001
miR‐144‐3p	6.04 ± 1.27	6.67 ± 1.15	1.55	.020	3.81 ± 1.72	5.15 ± 2.11	2.53	<.001	5.53 ± 1.67	6.31 ± 1.67	1.72	.001
miR‐17‐5p	2.28 ± 0.79	4.10 ± 0.99	3.54	<.001	0.98 ± 1.76	3.55 ± 1.15	5.92	<.001	4.34 ± 1.05	5.76 ± 1.03	2.66	<.001
miR‐20b‐5p	5.55 ± 0.75	7.05 ± 0.93	2.84	<.001	4.31 ± 1.85	6.65 ± 1.33	5.06	<.001	6.51 ± 0.95	7.88 ± 0.88	2.57	<.001
miR‐205‐5p	9.27 ± 1.26	10.09 ± 1.09	1.77	.014	7.94 ± 1.96	9.28 ± 2.00	2.53	<.001	9.02 ± 2.00	9.87 ± 1.40	1.80	.047
miR‐20a‐5p	4.34 ± 1.08	6.38 ± 1.09	4.12	<.001	5.86 ± 2.60	7.70 ± 1.87	3.56	<.001	5.14 ± 0.66	5.93 ± 0.61	1.74	.028

Abbreviations: FC, fold change; NCs, normal controls; NPC, nasopharyngeal carcinoma.

### 3.4 Correlation between miRNA expression levels and clinicopathologic features

**Figure 3:**
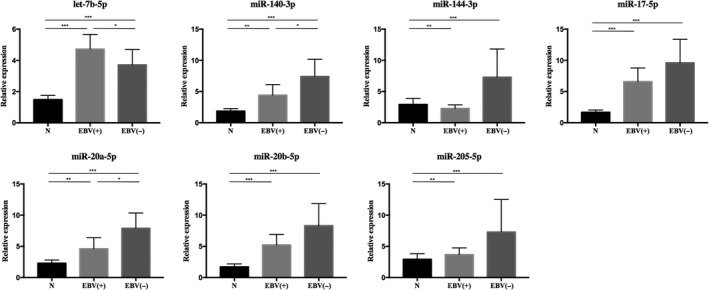
Expression levels of the identified plasma miRNAs in EBV‐positive and EBV‐negative NPC patients in comparison with normal controls. EBV(+): EBV‐positive, n = 92; EBV(−): EBV‐negative, n = 105; N: normal control, n = 189 (Y axis was presented as relative expression. Horizontal line: mean with 95% CI. *P < .05, ** *P* < .01, *** *P* < .001.)

Since NPC is highly correlated with EBV infection status, we further tried to explore miRNA expression difference in plasma among 189 NCs and EBV‐positive or EBV‐negative (confirmed by EBV‐DNA test) NPC patients from the 200 cases. As shown in Figure 3, all the seven miRNAs but miR‐144‐3p were significantly up‐regulated in EBV‐positive NPC patients compared to NCs. miR‐144‐3p were conversely down‐regulated with *P* < .05. Parallelly, significant up‐regulation of the seven plasma miRNAs were also observed in EBV‐negative NPC patients compared to NCs (*P* < .05). In addition, when we compared miRNA expression in plasma between EBV‐positive and EBV‐negative NPC patients, we found that the expression level of let‐7b‐5p was significantly higher in the former group while miR‐140‐3p and miR‐20a‐5p were much lower than in the latter group (*P* < .05). Besides EBV infection status, TNM stage and lymph node metastasis status of NPC were also taken into consideration. No significant difference was observed in miRNA expression levels for any of the seven miRNAs in plasma between early‐stage (Stage I or II) NPC patients and advanced (Stage III or IV) NPC patients (*P* > .05, Figure S1). So was the result of comparison between NPC patients with or without (N0/N1 vs N2/N3) distant lymph node metastasis (*P* > .05, Figure S2).

### 3.5 Diagnostic value of identified miRNA signature in plasma for NPC

ROC (receiver operating characteristic) curves were constructed and AUC (area under the ROC curve) values were calculated to explore the diagnostic value of the identified seven plasma miRNAs in discriminating NPC patients from NCs. When the training, testing, and external validation stages were combined, the AUCs were 0.762 (95% confidence interval (CI): 0.716‐0.808) for let‐7b‐5p, 0.622 (95% CI: 0.569‐0.676) for miR‐140‐3p, 0.619 (95% CI: 0.564‐0.673) for miR‐144‐3p, 0.734 (95% CI: 0.686‐0.782) for miR‐17‐5p, 0.640 (95% CI: 0.586‐0.693) for miR‐20a‐5p, 0.732 (95% CI: 0.684‐0.780) for miR‐20b‐5p and 0.633 (95% CI: 0.577‐0.688) for miR‐20‐5p, respectively (Figure S3).

**Figure 4:**
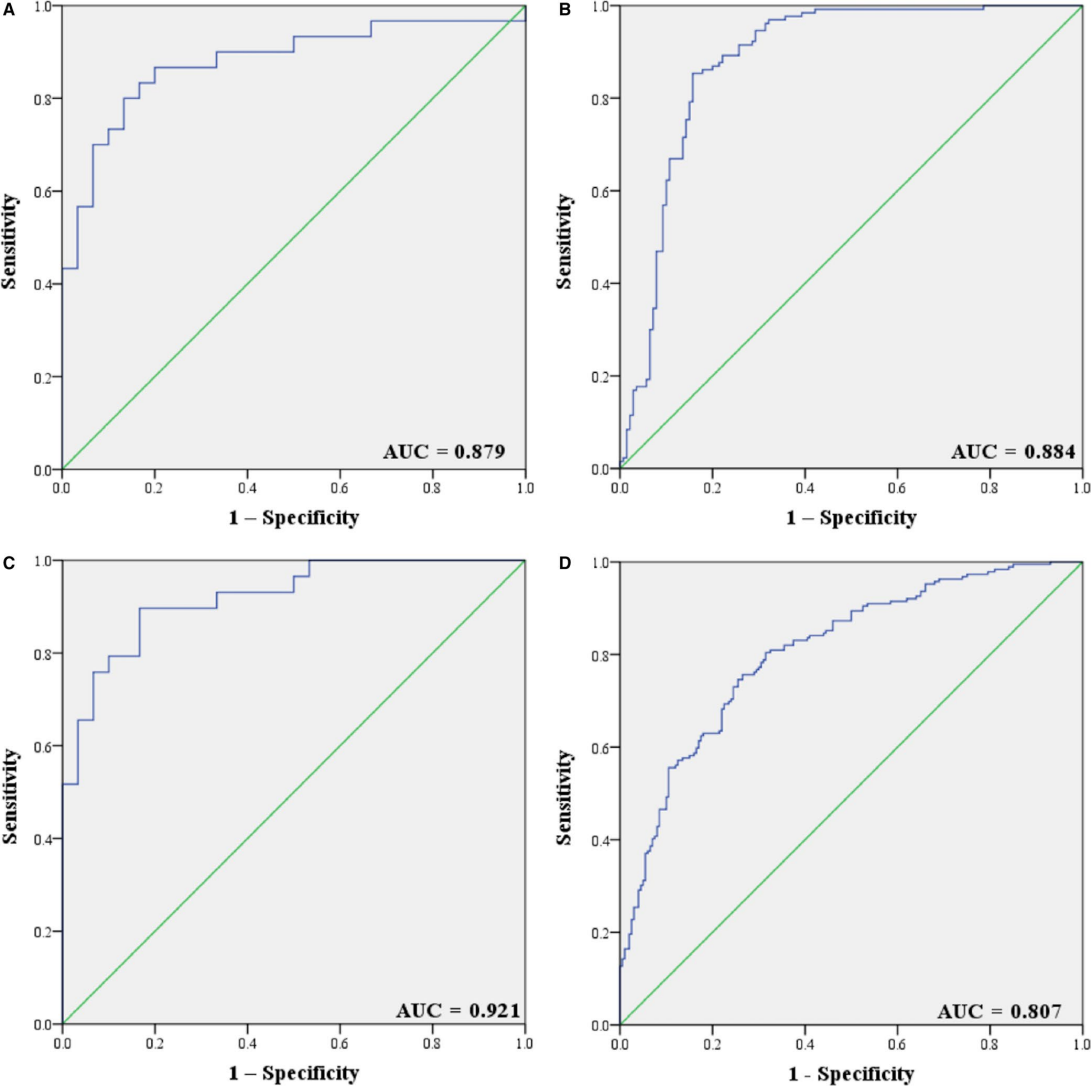
Receiver operating characteristic (ROC) curve analysis of the seven‐miRNA panel for NPC detection. A, training stage (30 NPC vs 30 NCs); (B) testing stage (140 NPC vs 130 NCs); (C) external validation stage (30 NPC vs 29 NCs); (D) combined three stages (200 NPC vs 189 NCs) (ROC curve: receiver‐operating characteristic curve; AUC: area under the ROC curve.)

In an attempt to enhance the diagnostic efficacy for NPC patients, we combined the identified miRNAs into a comprehensive panel using binary logistic regression analysis. A logistic regression statistical model that provide accurate discrimination of NPC patients from healthy people was developed: Logit(P) = −0.981 + 0.207 × let‐7b‐5p – 0.066 × miR‐140‐3p − 0.005 × miR‐144‐3p + 0.277 × miR‐17‐5p − 0.013 × miR‐20a‐5p − 0.004 × miR‐20b‐5p + 0.003 × miR‐205‐5p. The 7‐miRNA panel yielded AUCs of 0.879 (95% CI: 0.787‐0.971, Figure 4A), 0.884 (95% CI: 0.841‐0.927, Figure 4B), and 0.921 (95% CI: 0.854‐0.987, Figure 4C) for the training, testing, and external validation phases, respectively. When the data from three phases were combined, the AUC was 0.807 with the sensitivity and specificity being 0.735 and 0.757 when 0.42 was set as the cutoff value (Table S2). Compared with individual miRNAs, the 7‐miRNA panel performed better in discriminating NPC patients from NCs.

Diagnostic value of the 7‐miRNA panel in discriminating NPC patients with different clinical characteristics from NCs was also evaluated in combination of the three independent stages. The AUCs were 0.753 (95% CI: 0.664‐0.841), 0.789 (95% CI: 0.733‐0.846), and 0.829 (95% CI: 0.769‐0.889) for Stage II, III, and IV NPC patients in comparison with NCs (Figure S4). Similarly, the diagnostic performance of identifying EBV‐negative and EBV‐positive NPC patients from NCs remained fine. The corresponding AUCs were 0.827 (95% CI: 0.778‐0.876) for EBV‐negative patients and 0.823 (95% CI: 0.768‐0.877) for EBV‐positive patients (Figure S5).

### 3.6 Prognostic value of the miRNA signature for NPC

Cox regression and Kaplan‐Meier curve analyses were conducted to estimate the association between several clinical influence factors and OS (overall survival) rate. As shown in Table S3, in univariate Cox regression analysis, distant lymph node metastasis had significant association with worse OS for NPC patients (*P* < .05). However, none of the seven identified miRNAs showed close correlation with NPC prognosis (*P* > .05) (Figure S6).

### 3.7 miRNA expression in tissues

**Figure 5:**
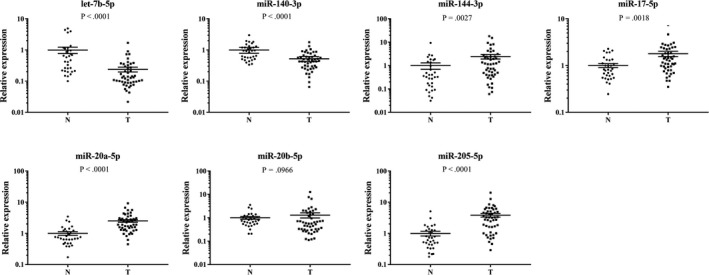
Expression levels of the seven miRNAs in 48 NPC tumor tissue and 32 healthy nasal mucosa tissue specimens (N: normal control; T: tumor; Horizontal line: mean with SEM.)

Expression levels of the identified seven miRNAs were also analyzed among 48 NPC tissue specimens and 32 nasal mucosa tissue specimens from healthy donors on the basis of qRT‐PCR. As shown in Figure 5, let‐7b‐5p and miR‐140‐3p were significantly down‐regulated in NPC tumor tissues, while miR‐144‐3p, miR‐17‐5p, miR‐20a‐5p, and miR‐205‐5p were significantly up‐regulated in NPC tissues compared to normal tissues (*P* < .05). No significant difference was observed for miR‐20b‐5p expression.

### 3.8 miRNA expression in plasma exosomes

For better understanding of the potential existing form of the identified plasma miRNAs, we further explored miRNA expression patterns in 32 NPC vs 32 NCs plasma‐derived exosomes samples by qRT‐PCR. None of the seven miRNAs showed expression difference with statistical significance in plasma exosomes (*P* > .05, Figure S7).

### 3.9 Bioinformatics analysis of identified miRNAs

**Figure 6:**
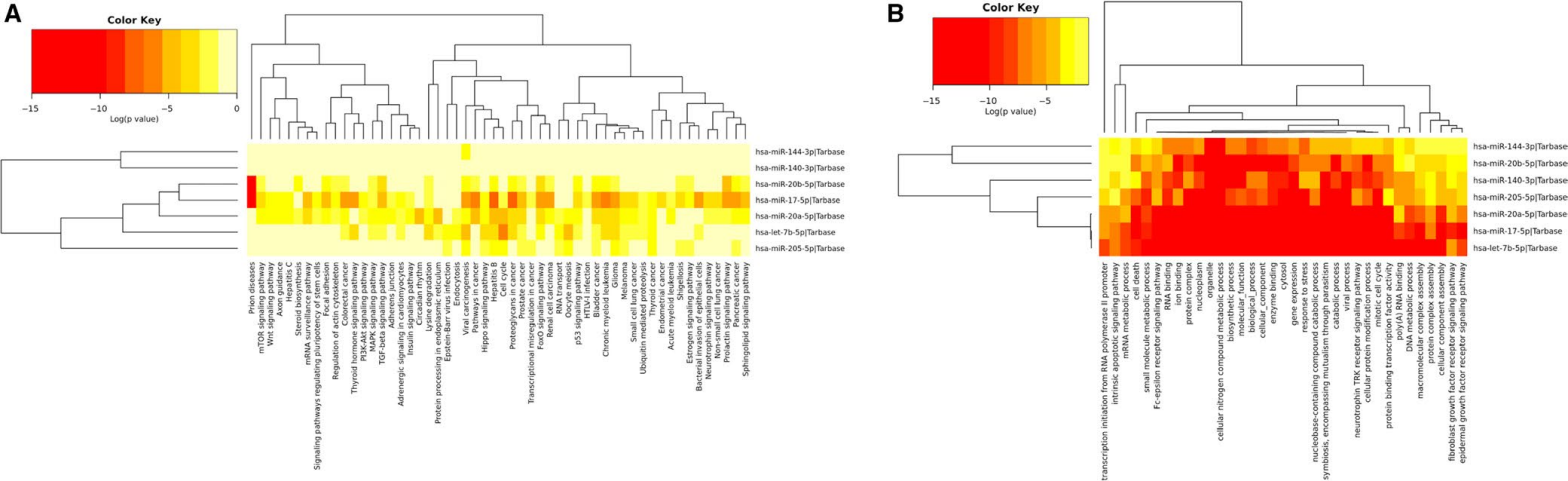
Heatmap of KEGG and GO pathway analyses of the identified seven miRNAs. A, KEGG (category union); (B) GO (category intersection) (KEGG: Kyoto Encyclopedia of Genes and Genomes; GO: Gene Ontology.)

DIANA‐miRPath v3.0 analysis of miRNA target genes and correlated‐pathways based on DIANA‐TarBase v7.0 database was conducted to decipher the potential function of each identified miRNA. According to Kyoto Encyclopedia of Genes and Genomes (KEGG) analysis, these miRNAs were involved in several tumor‐related pathways such as p53 signaling pathway, viral carcinogenesis, and FoxO signaling pathway. Gene Ontology (GO) category analysis identified several biological processes associated with these miRNAs (such as ion binding, cell death, cell cycle, and immune system process). The heatmaps and tables of miRNA target analysis are presented in Figure 6 and Table S4.

## 4 DISCUSSION

NPC, although with its gradually declining mortality rate due to tremendous advances in screening methods and treatment strategies, still poses great threat to residents in some specific regions or ethnic groups.^24^ NPC patients in their early stage with non‐metastatic diseases always have good response to local radiotherapy (especially intensity‐modulated radiotherapy [IMRT]).^25^ However, a number of NPC cases are asymptomatic until the disease develops to an advanced stage, which attributes to their poor prognosis.^26^ Since EBV infection is consistently correlated with NPC, quantification of cell‐free EBV DNA in plasma as well as detection of EBV‐based antibodies can serve to monitor the occurrence, development, prognostication, or treatment outcomes of the disease.^27^, ^28^, ^29^ Nevertheless, furthermore research is in need to confirm their clinical use and to discover novel non‐invasive biomarkers for disease surveillance. MiRNAs are closely implicated in tumor‐promotion or suppression activities and have been proved to be potential biomarkers for various cancers.^16^ For NPC, miRNA expression has been explored at different scopes by numerous studies which often concentrated on intracellular miRNAs in tumor tissues or EBV‐originated viral miRNAs.^30^, ^31^ However, still a few efforts have been made to decipher the miRNA expression traits in the plasma of NPC patients nor identify novel circulating miRNAs capable for NPC screening.

In this study, we conducted a comprehensive four‐stage investigation among 389 plasma samples from 200 NPC patients and 189 NCs on the basis of qRT‐PCR. In the initial screening stage, 31 differently expressed miRNAs with 25 up‐regulated and 6 down‐regulated were screened out by the Exiqon miRNA qPCR panel and transferred to further validation in the following three stages (training, testing, and external validation stages) by qRT‐PCR. Ultimately, seven miRNAs (let‐7b‐5p, miR‐140‐3p, miR‐144‐3p, miR‐17‐5p, miR‐20a‐5p, miR‐20b‐5p, and miR‐205‐5p) in plasma showed consistent trend of up‐regulation in NPC patients compared to NCs. We combined the seven miRNAs together and constructed a 7‐miRNA panel to strengthen the diagnostic capability of the identified miRNA signature. ROC curve analyses were conducted and the corresponding AUCs for the panel for the three independent stages were as high as 0.879, 0.884, and 0.921, respectively. When the three stages were combined, the AUC was 0.807, which showed credible diagnostic value for the panel to discriminate NPC patients from NCs.

Up to now, several studies have also focused on the discovery of circulating miRNAs as biomarkers for NPC diagnosis. For example, Xiong Liu et al once observed the increased levels of plasma miR‐16, 21, 24, and 155 in NPC patients within candidate miRNA lists from other literatures,^32^ while Xiao‐Hui Zheng et al reported the significant up‐regulation of plasma miR‐548q and miR‐483‐5p based on array analysis.^33^ These results lacked consistency between each other and also had limited overlap with our findings, probably because of different initial screening methods, varied subject sizes or sample handling means. In this study, we employed the Exiqon miRNA qPCR panel to perform miRNA profiling in 2 NPC plasma pools and 1 NC pool for the preliminary selection of candidate miRNAs. Compared to other array based platforms such as TaqMan assays, this qRT‐PCR based platform could have better sensitivity and linearity in the case of relative lower miRNA abundance in plasma samples, which to some extent ensured the reliability and comprehensiveness of our candidate miRNAs.^34^ For further validation, we enrolled a considerable amount of study subjects with all the NPC patients being untreated when blood samples were taken, which could minimize the influence of any treatment factors and thus reveal the true expression patterns of plasma miRNAs in NPC patients.

For better understanding of the underlying roles of these identified miRNAs in tumor activities, we further explored miRNA expression levels in 48 NPC tissue samples vs 32 normal tissues. As a result, miR‐144‐3p, miR‐17‐5p, miR‐20a‐5p, and miR‐205‐5p were up‐regulated in NPC tumor tissues as in plasma compared to normal tissues. The up‐regulated miRNAs in plasma might be originated from tumor cells.^35^ let‐7b‐5p and miR‐140‐5p showed the opposite tendency of down‐regulation. Such discrepancy of miRNA expression patterns between blood samples and tissue specimens have been similarly observed in a number of previous studies.^36^, ^37^ Circulating miRNAs can have totally different expression traits with those intracellular. Active or passive transport of miRNAs between tumor cells, tumor‐adjacent normal cells and tumor micro‐environment may be one possible explanation.^38^, ^39^ Moreover we suspect that miRNA expression changes in tissue just reflected local changes, but miRNA expression in blood circulation might be the epitome of systematic disease status. However, the exact mechanism remains unclear and still requires further investigation.

Although potential biomarkers of circulating miRNAs have been identified for NPC diagnosis, exploration of their function in NPC carcinogenesis and progression is still in its infancy. miR‐17‐5p and miR‐20a‐5p are members of the miR‐17‐92 cluster, and their oncogenic function such as cell cycle regulation has been confirmed with numerous evidence in various cancers.^40^, ^41^ In NPC, overexpressed miR‐17‐5p might promote tumor occurrence and proliferation via down‐regulating the expression of p21 protein (a cell cycle inhibitor),^42^ and according to Zhao et al, the significant up‐regulation of miR‐20a‐5p could promote resistance to radiotherapy for NPC patients via targeting the gene of neuronal PAS domain protein 2 (NPAS2) and regulating the Notch signaling pathways which were involved in cell proliferation, differentiation and apoptosis.^43^ For miR‐144‐3p and miR‐205‐5p, their tumor‐promoting roles in NPC have also been revealed by several studies. miR‐144‐3p was located in 17q11.2, the region often amplified in NPC patients.^44^ In NPC, miR‐144‐3p was discovered to promote tumor migration and invasion by down‐regulating the tumor suppressor gene phosphatase and tensin homolog (PTEN) and activating the PI3K/Akt pathway.^45^ miR‐205‐5p have complex roles of oncogenicity and anti‐oncogenicity in different cancers. But in NPC, according to Nie G et al, it functioned as tumor‐promotor via targeting tumor protein p53‐inducible nuclear protein 1.^46^ miR‐17‐5p could also modulate radio‐resistance of NPC through targeting PTEN.^47^ According to the previous study, let‐7 family could prevent tumor cells proliferation by suppressing c‐Myc expression in NPC and the dysregulation of miRNA let‐7 might be associated with the early formation of NPC.^48^ Evidence was still limited, but our findings might provide some hints for future investigation.

To supplement the diagnostic application of the identified signature, miRNA expression levels were further evaluated among NPC patients with different clinical characteristics. When EBV‐positive and EBV‐negative patients were analyzed separately, we could observe the opposite trait of down‐regulation for plasma miR‐144‐3p in EBV‐positive patients in comparison with NCs. Interestingly, EBV‐positive and EBV‐negative NPC patients could exhibit different miRNA expression patterns in plasma. Among the seven miRNAs, expression levels of miR‐140‐3p and miR‐20a‐5p were lowered while let‐7b‐5p was increased with the presence of EBV infection for NPC cases. The results of this primary exploration suggested that active EBV infection might alter the miRNA expression patterns in plasma for NPC patients. Several previous studies revealed that EBV infection could influence the expression levels of certain miRNAs (eg miR‐146a and miR‐155) to promote NPC development.^49^ However, none of these studies had focused on the crosstalk between circulating miRNA expression and EBV infection status in NPC. In this study, such a phenomenon observed in different subgroups was still preliminary but might give a hint to future investigation. Besides EBV infection status, miRNA expression levels in plasma were also evaluated between patients in advanced stage (Stage III or IV) and early stage (Stage I or II), as well as patients with and without distant lymph node metastasis. Although no significant difference was observed, the 7‐miRNA panel could still do well to discriminate each group of fine‐classified patients from NCs. In addition, Cox regression analysis and Kaplan‐Meier curves were performed for survival analysis. It seemed that none of the seven identified miRNAs could actually measure the clinical prognosis of NPC patients. The results indirectly indicated that most NPC patients can respond well to timely treatment, which apparently prolong patients’ OS in clinical practice.

Besides plasma and tissue samples, miRNA expression levels were further analyzed in plasma‐derived exosomes from 32 NPC patients and 32 NCs. Exosomes, one of the smallest extracellular vesicles secreted by many cell types, have shown to carry a variety of molecules including miRNAs.^50^ Exosomal miRNAs can form potential biomarkers for various cancers and may help improve comprehension over some unexplained cancer behaviors. However, in our study, no significant difference was observed for any of the seven up‐regulated plasma miRNAs in plasma‐derived exosomes between NPC and NCs. It is notable that besides exosomes, the majority of miRNAs are loaded to the fundamental extracellular carrier—Agonaute2 (Ago2) proteins. According to Arroyo JD et al, five miRNAs in our study (let‐7b‐5p, miR‐140‐3p, miR‐144‐3p, miR‐20a‐5p and miR‐20b‐5p; the other two not well detected) were not encapsulated in exosomes but independently co‐purified with Ago2 ribonucleoprotein complex in plasma.^51^ It could be one of the credible explanations of discrepant miRNA expression traits between plasma and exosomes.

Taken together, we identified a 7‐miRNA signature in plasma for NPC detection. Although there will be still a long way to go for virtual clinical use, giving consideration to its convenience and low health impact, the miRNA panel can be combined with some traditional strategies to assist disease screening and benefit clinical outcomes of NPC patients in the near future.

## CONFLICT OF INTERESTS

The authors declare that they have no conflict of interest.

## ETHICS APPROVAL AND CONSENT TO PARTICIPATE

All procedures were approved by Institutional Review Boards of the First Affiliated Hospital of Nanjing Medical University and Jiangsu Cancer Hospital. Written informed consent was obtained from patients involved in the study.
