# Supplementary material for: Identification of a 7‐microRNA signature in plasma as promising biomarker for nasopharyngeal carcinoma detection
Source: Cancer Med. 2019 Dec 19;9(3):1230–41. doi: 10.1002/cam4.2676 (PMC6997065; doi:10.1002/cam4.2676)

| **MiRNA** | **Fold change** | | |
| --- | --- | --- | --- |
|  | **Pool 1** | **Pool 2** | **Mean fold** |
| **let-7b-5p** | 1.60 | 1.71 | 1.66 |
| **miR-10b-5p** | 2.22 | 1.62 | 1.92 |
| **miR-130a-3p** | 2.05 | 1.96 | 2.00 |
| **miR-140-3p** | 1.77 | 1.61 | 1.69 |
| **miR-144-3p** | 2.86 | 2.04 | 2.45 |
| **miR-17-5p** | 1.52 | 1.83 | 1.68 |
| **miR-20b-5p** | 2.90 | 1.71 | 2.31 |
| **miR-320c** | 1.66 | 2.22 | 1.94 |
| **miR-454-3p** | 2.96 | 1.88 | 2.42 |
| **miR-660-5p** | 1.81 | 1.88 | 1.85 |
| **miR-877-5p** | 1.91 | 1.59 | 1.75 |
| **miR-103a-3p** | 2.05 | 1.64 | 1.85 |
| **miR-141-3p** | 1.94 | 4.06 | 3.00 |
| **miR-144-5p** | 2.89 | 3.26 | 3.08 |
| **miR-200a-3p** | 2.53 | 8.93 | 5.73 |
| **miR-205-5p** | 6.10 | 7.65 | 6.88 |
| **miR-20a-5p** | 2.47 | 1.64 | 2.06 |
| **miR-34a-5p** | 2.38 | 4.25 | 3.32 |
| **miR-363-3p** | 1.55 | 1.79 | 1.67 |
| **miR-375** | 4.33 | 4.63 | 4.48 |
| **miR-451a** | 1.89 | 1.53 | 1.71 |
| **miR-483-5p** | 1.98 | 2.49 | 2.24 |
| **miR-497-5p** | 12.79 | 1.90 | 7.35 |
| **miR-7-5p** | 5.13 | 2.61 | 3.87 |
| **miR-16-5p** | 1.95 | 2.07 | 2.01 |
| **miR-1** | -2.30 | -2.82 | -2.56 |
| **miR-326** | -17.95 | -18.03 | -17.99 |
| **miR-376a-3p** | -12.95 | -14.94 | -13.95 |
| **miR-485-3p** | -26.16 | -53.33 | -39.75 |
| **miR-136-3p** | -4.04 | -19.46 | -11.75 |
| **miR-199a-5p** | -98.08 | -117.13 | -107.61 |

**Table S1.** Candidate miRNAs identified in the screening stage.

**Table S3.** The relationship between OS and clinical factors for NPC

| **Variables** | **Univariate analysis** | | |
| --- | --- | --- | --- |
|  | **HR (95%CI)** | **P value** |  |
| Gender (male VS. female) | 5.273(0.702,39.621) | 0.106 |  |
| Age (>60 VS. ≤60) | 1.703(0.639,4.538) | 0.287 |  |
| TNM stage (III+IV VS. I+II) | 5.713(0.760,42.948) | 0.090 |  |
| T (3+4 VS. 1+2) | 2.213(0.728,6.724) | 0.161 |  |
| **N (2+3 VS. 0+1)** | **3.194(1.198,8.514)** | **0.020** |  |
| EBV (positive VS. negative) | 1.906(0.738,4.921) | 0.182 |  |
| let-7b-5p (≥median VS. <median) | 0.748(0.295,1.894) | 0.541 |  |
| miR-140-3p (≥median VS. <median) | 0.961(0.381,2.420) | 0.932 |  |
| miR-144-3p (≥median VS. <median) | 2.046(0.768,5.453) | 0.152 |  |
| miR-17-5p (≥median VS. <median) | 1.195(0.472,3.029) | 0.707 |  |
| miR-20b-5p (≥median VS. <median) | 0.955(0.379,2.406) | 0.922 |  |
| miR-205-5p (≥median VS. <median) | 1.587(0.615,4.095) | 0.339 |  |
| miR-20a-5p (≥median VS. <median) | 1.242(0.490,3.147) | 0.648 |  |
| predicted point (≥median VS. <median) | 0.988(0.371, 2.643) | 0.981 |  |

NPC: nasopharyngeal carcinoma; OS: overall survival; HR: hazard ratio; CI: confidence interval; T: tumor topography; N: lymph node; EBV: Epstein-Barr Virus.

**Figure S1.** Expression levels of the seven identified plasma miRNAs among NPC patients at different stages (stage I/II versus stage III/IV). (Y axis represents the relative expression (2^-ΔΔCt^); Horizontal line: mean with 95% CI.)


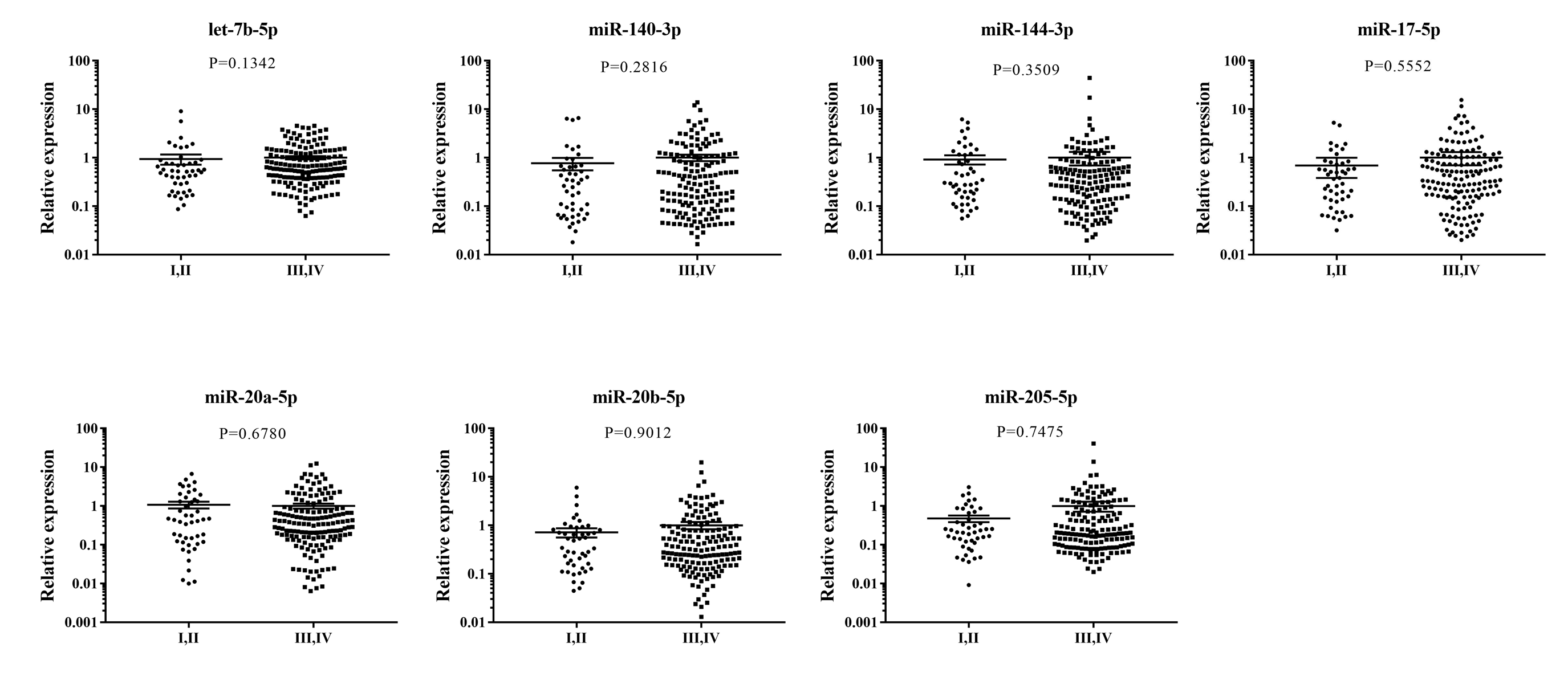


**Figure S2.** Expression levels of the identified plasma miRNAs among NPC patients with different lymph node metastasis conditions (N0/N1 versus N2/N3). (Y axis represents the relative expression (2^-ΔΔCt^); Horizontal line: mean with 95% CI.)


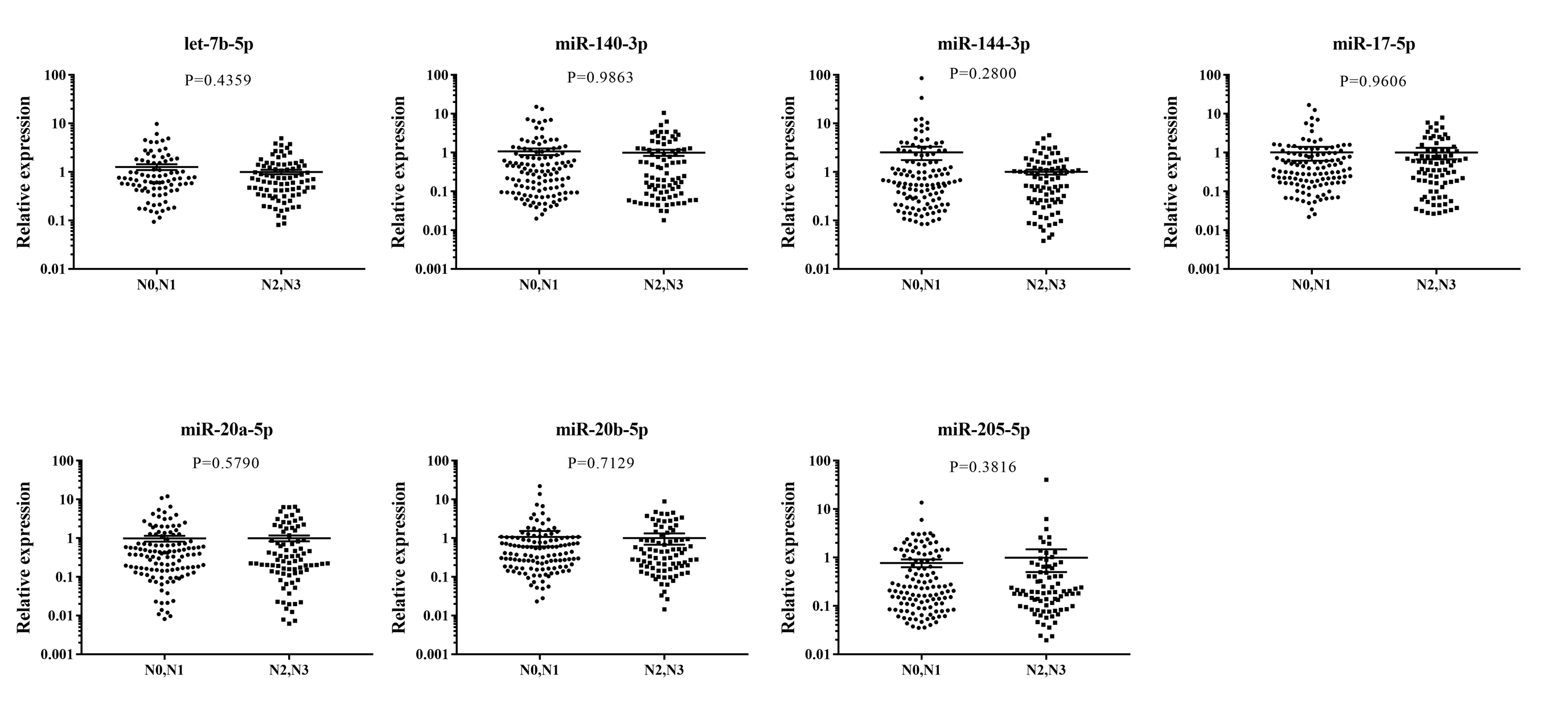


**Figure S3.** ROC curves of the seven plasma miRNAs for NPC detection in combined three cohorts of training, testing and external validation phases (200 NPC VS. 189 NCs). (ROC curve: receiver-operating characteristic curve; AUC: area under the ROC curve.)


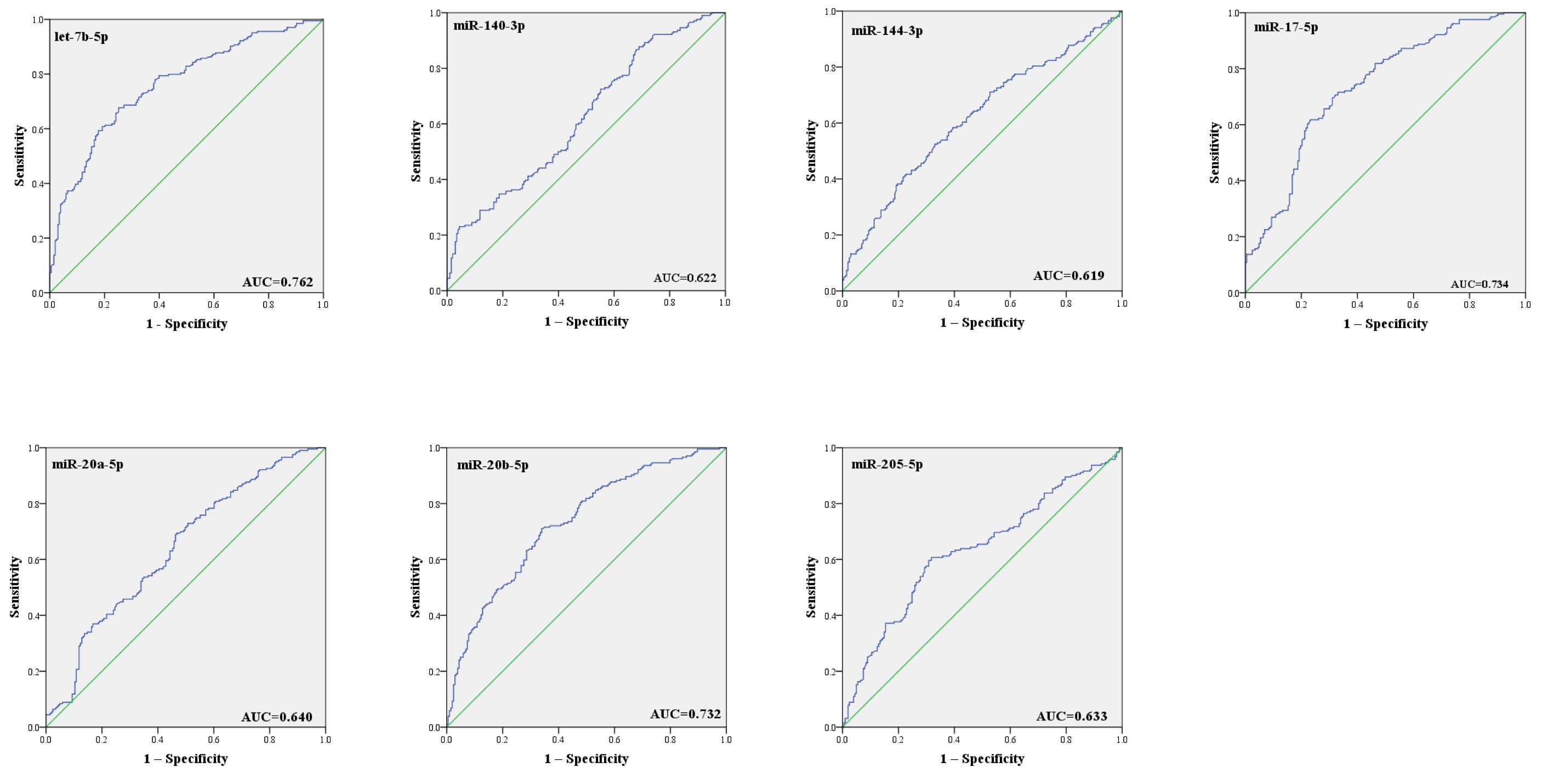


**Figure S4.** ROC curve analysis of the seven-miRNA panel in plasma for the detection of NPC patients at different stages in comparison with NCs. A: Stage II VS. NCs; B: Stage III VS. NCs; C: Stage IV VS. NCs. (ROC curve: receiver-operating characteristic curve; AUC: area under the ROC curve.)


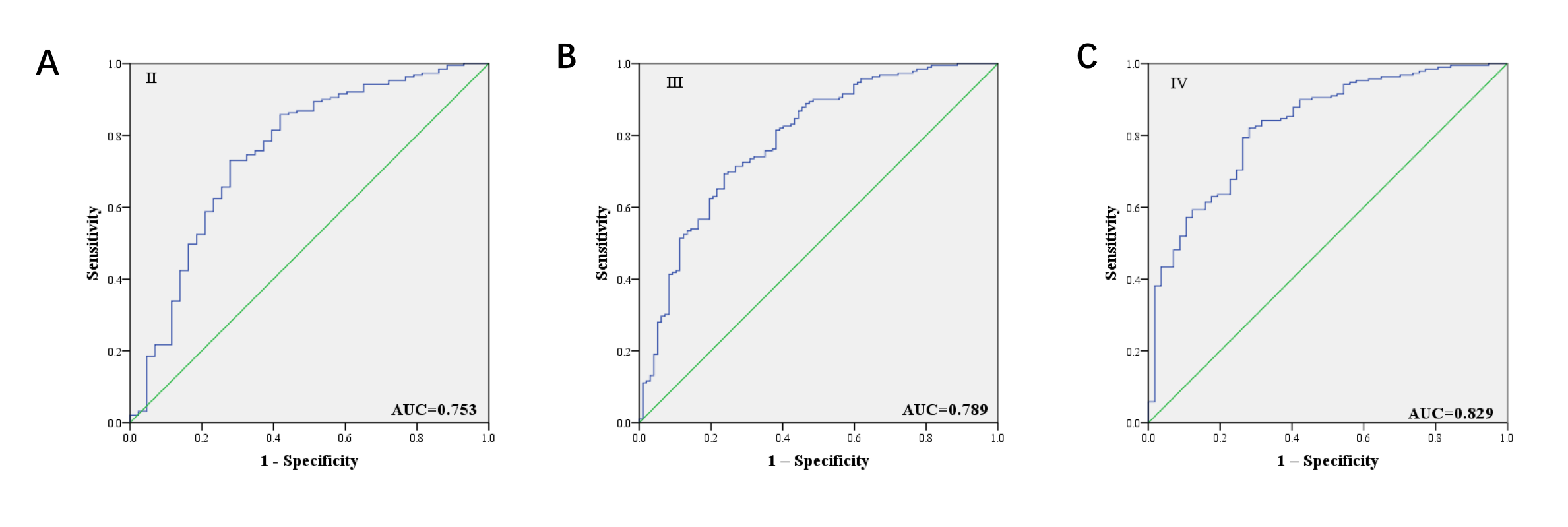


**Figure S5.** ROC curve analysis of the seven-miRNA panel in plasma for the detection of EBV-negative (A) or EBV-positive (B) NPC patients in comparison with NCs. (ROC curve: receiver-operating characteristic curve; AUC: area under the ROC curve.)


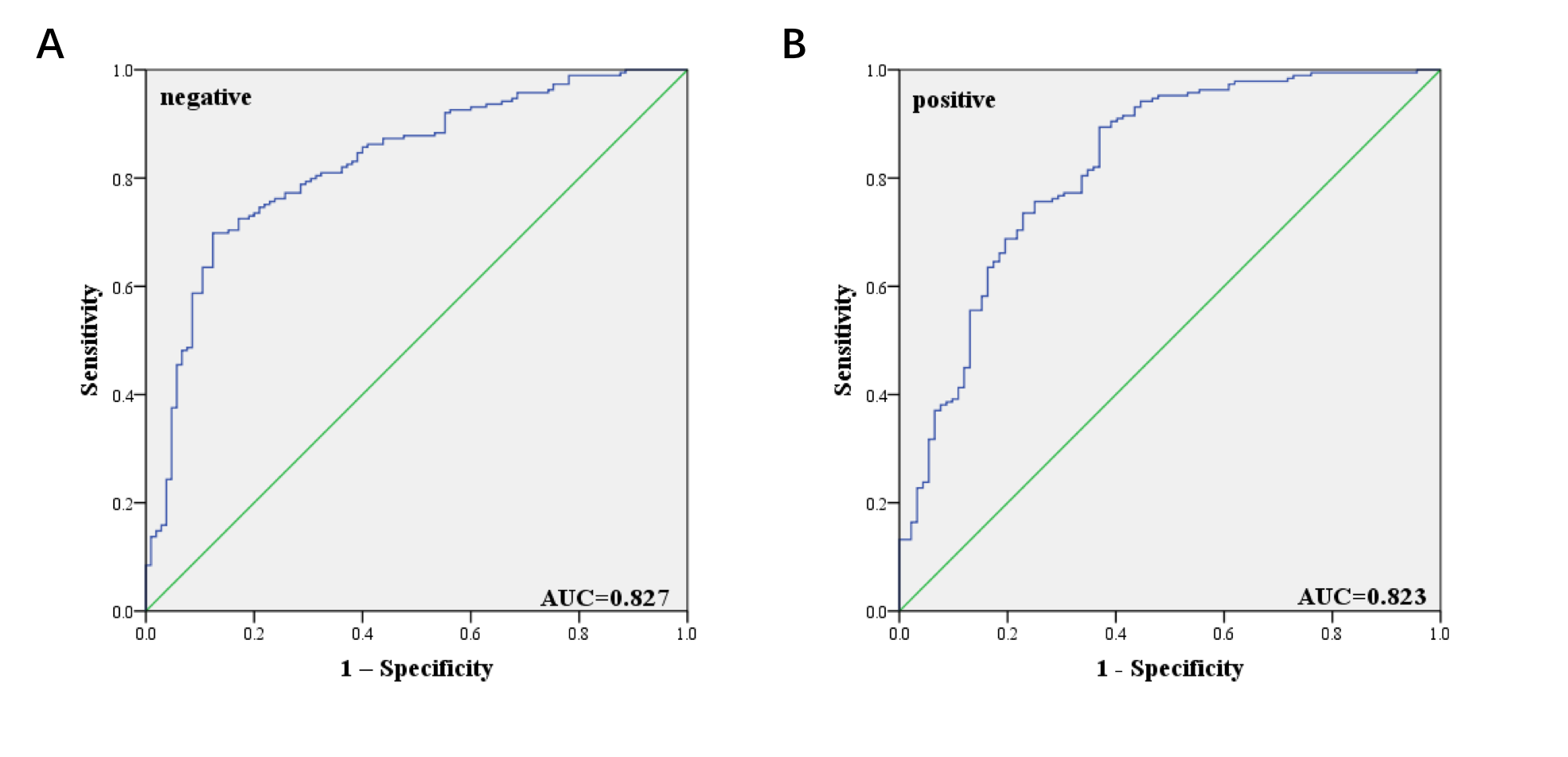


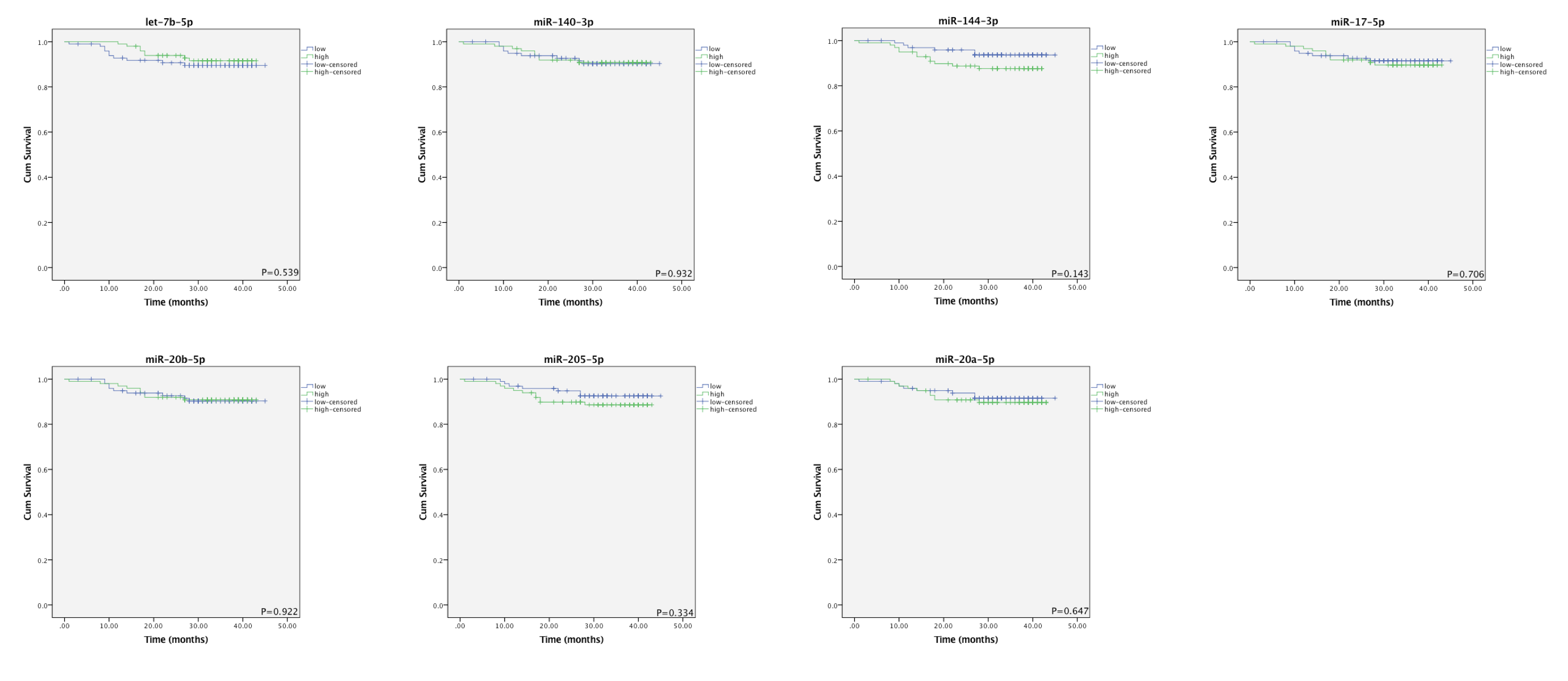
**Figure S6.** Kaplan–Meier curves estimating the association of the five identified miRNAs and the overall survival of NPC patients.

**Figure S7.** Expression levels of the five miRNAs in plasma-derived exosomes (32 NPC VS. 32 NCs). (N: normal control; T: tumor; Y axis represents the relative expression (2^-ΔΔCt^); Horizontal line: mean with SEM.)


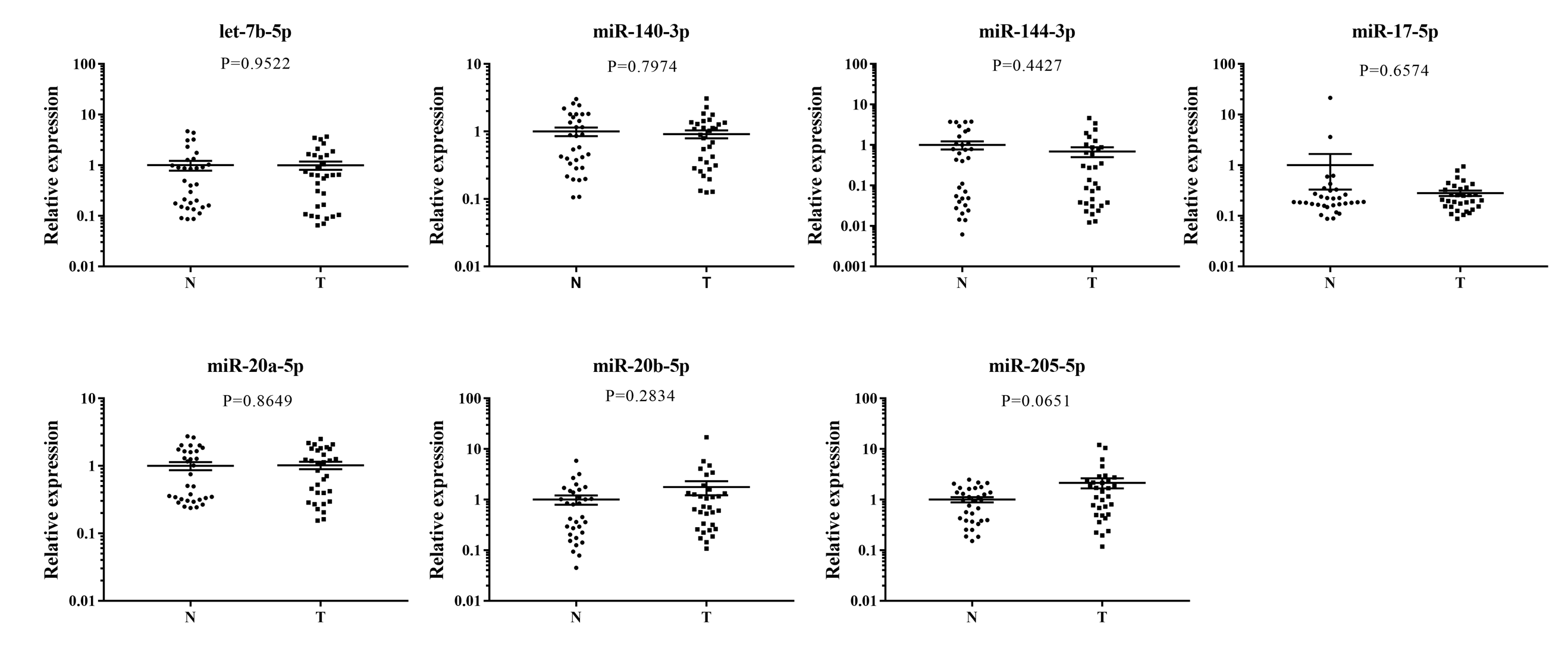

Supplement: Supplementary file 3 [file CAM4-9-1230-s003.docx]
